# How to Burn Coal without an Excess of Smoke

**Published:** 1908-03

**Authors:** 


### HOW TO BURN COAL WITHOUT AN EXCESS OF SMOKE.

Atlanta, unfortunately, has the distinction of being classed among the most smoky cities of this country, and we think that with a little care in the management of her furnaces, factories and power plants, this evil could be considerably abated. Smokeless combustion involves principles now well known, although not generally applied, in spite of the proven fact that it may be attained not only without additional cost, but really at a saving. The appearance of black smoke is a signal of incomplete combustion, and means not only that there is a loss of fuel in the soot particles, but to a much greater extent in the escape of gases which should have been utilized for heat. Some of these gasses that escape are identical with the gas we use in our homes for lighting and cooking purposes. If every manufacturer realized the actual loss in the dense black smoke which he sees emitted from the tall smoke stacks, we cannot believe he would view the matter so complacently. Of course the defective setting of some furnaces renders complete combustion a practical impossibility, but the city should make a greater effort to have furnaces properly repaired, where the defects can be corrected at a reasonable cost, and see to it that the firing be managed more carefully. The installation of new furnaces, when large, should be supervised by an expert with the abatement of this disagreeable feature in view.

The so-called smoke consumers are frequently non-effective, and, unfortunately, the high price of anthracite coal renders its use largely prohibitive, but these facts make it all the more important that the other and feasible measures receive more attention.

